# Association of Genes of the NO Pathway with Altitude Disease and Hypoxic Pulmonary Hypertension

**DOI:** 10.3390/jcm10245761

**Published:** 2021-12-09

**Authors:** Juliane Hannemann, Patricia Siques, Lena Schmidt-Hutten, Julia Zummack, Julio Brito, Rainer Böger

**Affiliations:** 1 Institute of Clinical Pharmacology and Toxicology, University Medical Center Hamburg-Eppendorf, 20246 Hamburg, Germany; l.schmidt-hutten@uke.de (L.S.-H.); julia.zummack@web.de (J.Z.); boeger@uke.de (R.B.); 2 Institute DECIPHER, German-Chilean Institute for Research on Pulmonary Hypoxia and Its Health Sequelae, 20246 Hamburg, Germany and Iquique 1100000, Chile; psiques@tie.cl (P.S.); jbritor@tie.cl (J.B.); 3 Institute of Health Studies, Universidad Arturo Prat, Iquique 1100000, Chile

**Keywords:** high altitude, single nucleotide polymorphisms, asymmetric dimethylarginine (ADMA), DDAH, arginase, nitric oxide synthase, echocardiography, chronic intermittent hypoxia

## Abstract

Chronic intermittent hypoxia leads to high-altitude pulmonary hypertension, which is associated with high asymmetric dimethylarginine (ADMA), an endogenous inhibitor of nitric oxide synthesis. Therefore, we aimed to understand the relation of single nucleotide polymorphisms in this pathway to high-altitude pulmonary hypertension (HAPH). We genotyped 69 healthy male Chileans subjected to chronic intermittent hypoxia. Acclimatization to altitude was determined using the Lake Louise Score and the presence of acute mountain sickness. Echocardiography was performed after six months in 24 individuals to estimate pulmonary arterial pressure. The minor allele of dimethylarginine dimethylaminohydrolase (DDAH)1 rs233112 was associated with high-baseline plasma ADMA concentration, while individuals homozygous for the major allele of DDAH2 rs805304 had a significantly greater increase in ADMA during chronic intermittent hypoxia. The major allele of alanine glyoxylate aminotransferase-2 (AGXT2) rs37369 was associated with a greater reduction of plasma symmetric dimethylarginine (SDMA). Several genes were associated with high-altitude pulmonary hypertension, and the nitric oxide synthase (NOS)3 and DDAH2 genes were related to acute mountain sickness. In conclusion, DDAH1 determines baseline plasma ADMA, while DDAH2 modulates ADMA increase in hypoxia. AGXT2 may be up-regulated in hypoxia. Genomic variation in the dimethylarginine pathway affects the development of HAPH and altitude acclimatization.

## 1. Introduction

Chronic exposure to global hypoxia, e.g., at high altitude, may cause generalized hypoxic pulmonary vasoconstriction, leading to pulmonary arterial hypertension [1,2]. This pulmonary vasoconstriction is partly caused by diminished endothelium-dependent, nitric oxide (NO)-mediated vasodilation, a molecular response that is in sharp contrast to up-regulation of NO synthase in the systemic arterial endothelium in hypoxia [3,4,5].

We previously demonstrated that asymmetric dimethylarginine (ADMA), an endogenous inhibitor of NO synthesis, is up-regulated in chronic intermittent hypoxia (CIH), a condition affecting large numbers of individuals living at sea level and working at high altitude, e.g., in the Chilean mining areas in the Andes [1]. These data are in line with those derived from animal models of chronic hypoxia [6,7]. In a prospective study among 123 healthy males, we showed that ADMA not only is elevated during six months of chronic intermittent hypoxia, but also that elevated ADMA concentration at baseline is a predictor of high-altitude pulmonary hypertension incidence [8].

ADMA is formed during post-translational protein methylation by protein arginine N-methyltransferases (PRMTs) [9]. It is primarily metabolized by dimethylarginine dimethylaminohydrolases (DDAH1 and DDAH2); alanine glyoxylate aminotransferase-2 (AGXT2) also metabolizes ADMA and symmetric dimethylarginine (SDMA) [10,11].

In humans, it is known that single nucleotide polymorphisms may affect both basal expression of proteins and the transcriptional and translational response to environmental stimuli. Multiple studies showed associations of single nucleotide polymorphisms in the NOS3 gene (that encodes for endothelial NOS) with vasospasm, hypertension, and cardiovascular death [12]. Polymorphisms in the NOS3, DDAH1, DDAH2, and AGXT2 genes have previously been shown to relate to NO-mediated vascular function, hypertension, and mortality in a range of different patient populations [13,14,15]. 

It was the objective of this study to assess whether genetic variation in the L-arginine—dimethylarginine—NO pathway modulates the individual response to chronic intermittent hypobaric hypoxia. Therefore, we selected 16 single nucleotide polymorphisms in NOS3, DDAH1, DDAH2, AGXT2, ARG1, ARG2, and PRMT1 genes and studied their associations with dimethylarginine concentrations and the incidence of high-altitude pulmonary hypertension as well as acclimatization to high altitude.

## 2. Materials and Methods

### 2.1. Study Participants and Protocol

Whole blood samples for DNA extraction were available from 69 healthy male Chilean army draftees who had never been exposed to high altitude before, during exposure to six months of chronic intermittent hypobaric hypoxia [8]. After the baseline investigation, the study participants were followed during a regimen of five days at high altitude (3550 m) followed by two days of recovery at sea level during six months. Written informed consent was obtained from all study participants before the start of the study. This observational study was approved by the Ethical Committee of Universidad Arturo Prat, Iquique, Chile.

We extracted DNA from blood samples collected at baseline; venous blood samples for the measurement of ADMA and SDMA concentrations were taken at baseline (sea level) and at six months (high altitude). Haematocrit and haemoglobin were measured on the day of blood withdrawal using an automatic haematological counter (Cell-dyn 3700^®^, Tecnigen, Santiago, Chile). Blood was centrifuged and EDTA plasma kept frozen at −20 °C until analysis of dimethylarginines. ADMA and SDMA were quantified using a validated liquid chromatography—tandem mass spectrometry method (LC-MS/MS) [16].

Blood oxygen saturation (SaO_2_) was measured with a pulse oximeter (POX050, Mediaid^®^, Cerritos, CA, USA). Acute mountain sickness was diagnosed clinically when headache and one or more other typical symptoms occurred (i.e., nausea or vomiting, insomnia, dizziness, lassitude, or fatigue) and a Lake Louise Score [17] of >3 was achieved. The level of acclimatization to high altitude was assessed by a combination of acute mountain sickness and SaO_2_: Good acclimatization was defined as absence of acute mountain sickness plus SaO_2_ > 89% and poor acclimatization was defined as presence of acute mountain sickness and/or SaO_2_ < 89%. After six months of chronic intermittent hypoxia, echocardiography was performed at high altitude in a subgroup of 24 study participants due to limited availability of technical resources [8]. Subjects undergoing echocardiography were selected to comprise both individuals with good (*n* = 10) and with poor acclimatization status (*n* = 14). A detailed description of the analysis of echocardiography measurements was previously published [8]. An estimated mPAP ≥ 30 mm Hg was used to define the presence of high-altitude pulmonary hypertension to account for the generally higher pulmonary arterial pressure at high altitude, as recommended by Leon-Velarde et al. [18]. 

### 2.2. Selection of Single Nucleotide Polymorphisms (SNPs)

**Figure 1:**
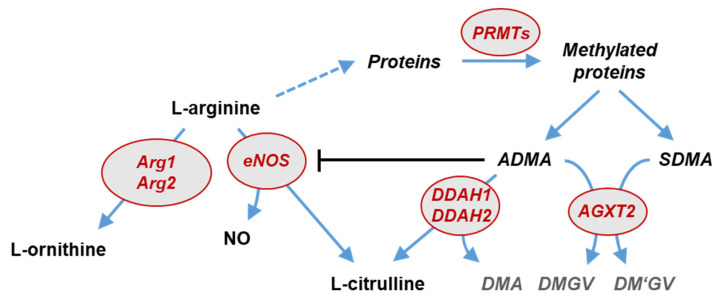
Schematic representation of the L-arginine—dimethylarginine—nitric oxide pathway. L-arginine is the substrate for endothelial NO synthase and arginases, resulting in the formation of NO and L-citrulline or L-ornithine, respectively. L-arginine residues within specific proteins are subject to methylation by protein arginine N-methyltransferases (PRMTs). After protein hydrolysis, asymmetric (ADMA) and symmetric dimethylarginine (SDMA) are released. ADMA is a competitive inhibitor of nitric oxide synthases (NOS). ADMA, but not SDMA, is degraded by dimethylarginine dimethylaminohydrolases (DDAH1 and DDAH2) into L-citrulline and dimethylamine (DMA). Both dimethylarginines may be cleaved by an alternative pathway through alanine glyoxylate aminotransferase 2 (AGXT2), resulting in the formation of symmetric or asymmetric dimethylguanidinovaleric acid (DMGV and DM’GV). Genes in which single nucleotide polymorphisms were studied in the present study are highlighted in red.

Single nucleotide polymorphisms were selected based upon an extensive search in the PubMed and NCBI SNP databases for publications linking single nucleotide polymorphisms in DDAH1, DDAH2, ARG1, ARG2, AGXT2, NOS3, and PRMT1 to endothelial function, NO synthesis, and the vascular response to hypoxia. These genes code for the key enzymes in the L-arginine—ADMA/SDMA—NO pathway (Figure 1) [4]. Subsequently, single nucleotide polymorphisms in the same gene were tested for linkage disequilibrium using the LDLink database of the National Cancer Institute, USA (https://ldlink.nci.nih.gov/ (accessed on 20 November 2021)) [19], and the list of single nucleotide polymorphisms was reduced to the best described one for this specific group when strong linkage disequilibrium was present; only polymorphisms that are not in strong linkage disequilibrium were included in the analyses.

### 2.3. DNA Isolation from Whole Blood

DNA from coagulated and centrifuged blood samples was isolated using QIAamp DNA Blood Mini Kit according to the manufacturer’s protocol (Qiagen, Hilden, Germany). Centrifugation steps were performed at room temperature and 20,000× *g*. DNA was eluted in a total volume of 200 µL. Subsequently, DNA was precipitated by addition of 1/10 vol. 3 M sodium acetate (pH 5.2) and 2.5 vol. absolute ethanol for further purification and concentration for SNP genotyping analyses. DNA was washed once with 70 % ethanol and resuspended in 20 µL H_2_0. DNA concentration was determined using a nanophotometer NP60 (Implen, Munich, Germany). 

### 2.4. Genotyping

Genotyping of single nucleotide polymorphisms was performed using single-tube human TaqMan SNP Genotyping Assays (Thermofisher, Waltham, MA, USA). Each reaction mix contained 10 ng template DNA, 0.5 µL specific TaqMan SNP Genotyping Assay and 5 µL of 2xTaqPath ProAmp™ Mastermix in a total reaction volume of 10 µL. The PCR was performed in a QuantStudio™ 5 Real-Time PCR System (Thermofisher, Waltham, MA, USA) using the following PCR program: pre-read (30 s, 60 °C), enzyme activation (5 min, 95 °C), 40 cycles of denaturation (5 s, 95 °C) and annealing (30 s, 60 °C), post-read (30 s, 60 °C). Allelic calls were identified by Quant Studio Design and Analysis Software (Thermofisher, Waltham, MA, USA).

### 2.5. Statistical Analyses

Statistical analyses were performed using SPSS (version 21; IBM Corporation, Armonk, NY, USA) and GraphPad Prism (version 6.01, GraphPad Software, San Diego, CA, USA). All variables were tested for normal distribution using the Kolmogorov–Smirnov test and for equal variances using the Levene test. Allele frequencies for the genes of interest (GOI) were compared to the Mixed American reference population or between groups using contingency tables and Fisher’s exact test. Definition of the major and minor allele of each SNP was made according to the allele distribution of the Mixed American reference population as given in the LDLink database [19]. Differences between groups were tested using either the nonparametric Mann–Whitney U test for two groups or the Kruskal–Wallis analysis of variance for more than two groups. Plasma ADMA and SDMA concentrations between genotypes were tested by ANOVA followed by the Scheffé f-test, and associations between allele frequencies versus clinical outcome were tested by the χ2 test using adjustment for multiple testing by Bonferroni-Holm [20]. Data are presented as median with 25th and 75th percentiles or as mean with standard error of the mean, as appropriate. For all tests, *p* < 0.05 was considered significant.

## 3. Results

### 3.1. Baseline Characteristics

The cohort consisted of 69 healthy Chilean males aged 18.4 ± 1.5 years, for whom complete data were available. Their physiological and biochemical baseline characteristics and baseline biomarker levels (Table 1) did not significantly differ from those of the larger group of individuals that we described before [8]. After six months of chronic intermittent hypoxia, haemoglobin and haematocrit consistently increased while SaO_2_ decreased (Table 1). Baseline ADMA and SDMA concentrations at sea level were (median (interquartile range)) 0.68 (0.60–0.75) µmol/L and 0.67 (0.59–0.75) µmol/L, respectively. ADMA increased to 0.72 (0.62–0.78) µmol/L after six months of chronic intermittent hypoxia (*p* = 0.024), while SDMA decreased to 0.58 (0.53–0.66) µmol/L (*p* = 0.008). 

### 3.2. Allele Frequencies of the Genetic Loci of Interest

The allele frequencies of the single nucleotide polymorphisms in this study did not significantly differ from the expected allele frequency based on the Mixed American reference population within the 1000 Genomes Project (Table 2), except for rs97548 in the PRMT1 gene, which had a slightly, but significantly higher than expected prevalence of the major allele in our cohort.

### 3.3. Association of Genotypes with Biomarker Concentrations

**Figure 2:**
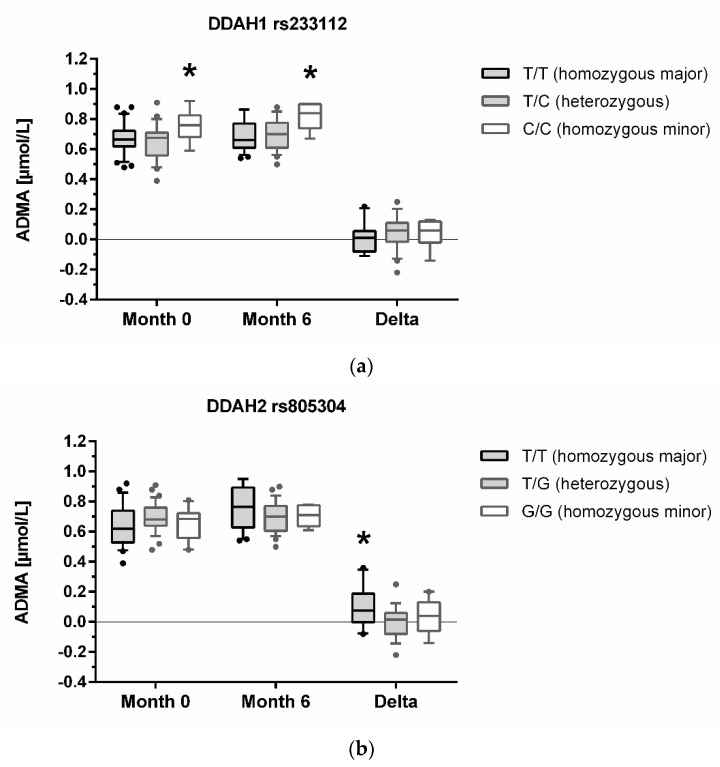

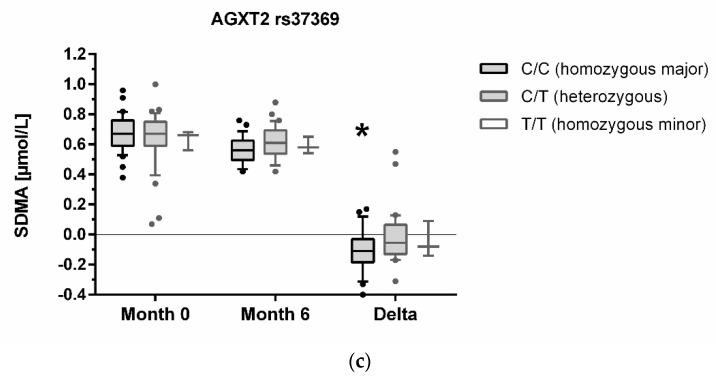
Dimethylarginine concentration with relation to DDAH1, DDAH2, and AGXT2 genotypes. Concentrations of ADMA at month 0 (baseline, sea level), month 6 (high altitude), and the increment of plasma ADMA concentration from month 0 to month 6 (Delta) are shown in different genotypes of DDAH1 rs233112 (**a**) and DDAH2 rs805304 (**b**). SDMA concentrations are shown in relation to AGXT2 rs37369 genotypes (**c**). Data are presented as median and interquartile range of *n* = 57–69 individuals, with the whiskers showing the 10th and 90th percentiles, respectively; outliers are plotted individually as dots. * denotes a statistically significant trend in differences of biomarker concentrations over genotypes (multiple comparisons ANOVA).

DDAH1 rs233112 and DDAH2 rs805304 genotypes were significantly associated with ADMA, and AGXT2 rs37369 genotypes were significantly associated with SDMA concentration. Individuals homozygous for the minor allele of DDAH1 rs2333112 had significantly higher median ADMA plasma concentrations, both at baseline and after six months of chronic intermittent hypoxia; however, the increment in ADMA during chronic intermittent hypoxia was not significantly different between genotypes (Figure 2a). By contrast, individuals homozygous for the major allele of DDAH2 rs805304 had a significantly greater increase in ADMA concentration after six months of chronic intermittent hypoxia, while absolute plasma concentrations of ADMA did neither differ between genotypes at baseline nor at 6 months (Figure 2b). SDMA plasma concentration showed a greater decrement after six months of chronic intermittent hypoxia in individuals homozygous for the major allele of AGXT2 rs37369, while median SDMA concentrations at baseline or six months did not differ significantly between genotypes (Figure 2c). There were no significant associations of any of the other genetic polymorphisms with these biomarkers.

### 3.4. Association of Genotypes with High-Altitude Pulmonary Hypertension and Altitude Acclimatization

**Figure 3:**
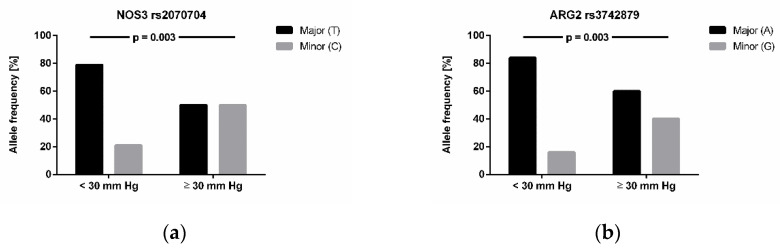

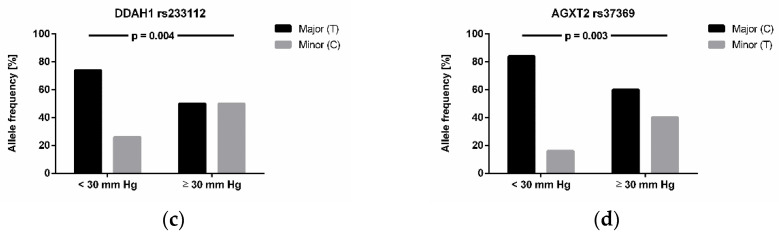
Relationship of genotypes of (**a**) NOS3 rs2070704, (**b**) ARG2 rs3742879, (**c**) DDAH1 rs233112, and (**d**) AGXT2 rs37369 with high-altitude pulmonary hypertension. Mean pulmonary arterial pressure was estimated by echocardiography after 6 months of chronic intermittent hypoxia in *n* = 24 individuals. Allele frequencies were analysed in individuals with (i.e., mPAP ≥ 30 mm Hg) or without high-altitude pulmonary hypertension (i.e., mPAP < 30 mm Hg). Data given are relative allele frequencies of the genes indicated. *p* values indicate significances in χ2 test after adjustment for multiple testing.

**Figure 4:**
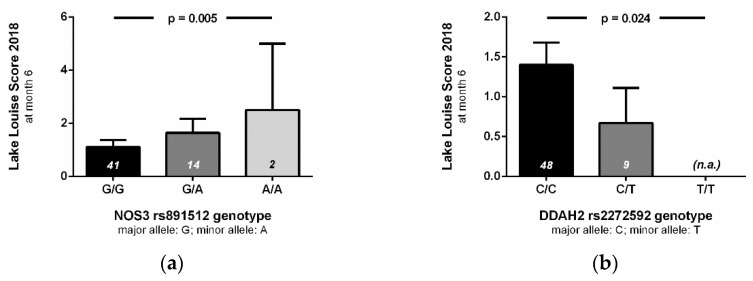
Relationship of genotypes with high-altitude acclimatization. Lake Louise Score (2018) values assessed at month 6 of chronic intermittent hypobaric hypoxia are given in relation to genotypes of the NOS3 rs891512 (**a**) and the DDAH2 rs2272592 single nucleotide polymorphism (**b**). Data are given as mean ± S.E.M. of *n* = 57 individuals. *p* values denote the results of multivariable-adjusted regression analysis.

We next tested the association of single nucleotide polymorphisms with high-altitude pulmonary hypertension (*n* = 24) and with acclimatization status to high altitude (*n* = 69). We found four genes to be significantly associated with high-altitude pulmonary hypertension (i.e., estimated mPAP ≥ 30 mm Hg) after adjustment for multiple testing (Figure 3): NOS3 rs2070744 (*p* = 0.003), ARG2 rs3742879 (*p* = 0.003), DDAH1 rs233122 (*p* = 0.004), and AGXT2 rs37369 (*p* = 0.003). NOS3 rs891512 was significantly associated with acclimatization status of the study participants after six months of chronic intermittent hypoxia (*p* = 0.001). Likewise, this same NOS3 polymorphism was significantly associated with Lake Louise Score at month 6 (*p* = 0.005), while the two other NOS3 polymorphisms (rs1799983 and rs2070744) were not associated with Lake Louise Score at month 6 (*p* = ns). DDAH2 rs2272592 also showed a significant association with Lake Louise Score at month 6 (*p* = 0.024; Figure 4). Interestingly, none of the single nucleotide polymorphisms analysed in this study was associated with Lake Louise Score at month 0, i.e., during first-time exposure to altitude.

## 4. Discussion

The present study has four major findings. First, single nucleotide polymorphisms of the DDAH1 gene are associated with baseline ADMA concentration. Secondly, the extent of increase of ADMA during chronic intermittent hypoxia is associated with single nucleotide polymorphisms in the DDAH2 gene. Thirdly, we observed a significant decrease in SDMA concentration during chronic intermittent hypoxia in individuals homozygous for the major allele of AGXT2 rs37369, while carriers of one or two minor alleles had no significant change in SDMA concentration. Finally, there were multiple associations of genes of the L-arginine—dimethylarginine—NO pathway with high-altitude pulmonary hypertension and acute mountain sickness.

We have previously observed an association between baseline ADMA concentration and incidence of high-altitude pulmonary hypertension after six months of chronic intermittent hypoxia [8]. Furthermore, hypoxic pulmonary arterial endothelial cells showed lower DDAH1 and DDAH2 mRNA and protein expression, lower DDAH activity, and higher ADMA concentration than normoxic cells [24]. In rats exposed to chronic hypobaric hypoxia, DDAH activity decreased and ADMA concentration in lung tissue increased [7]. However, DDAH1 knockout mice exposed to chronic hypoxia show the same extent of right ventricular hypertrophy as their wildtype littermates [6]. In line with this, single nucleotide polymorphisms in DDAH1 known to be associated with impaired DDAH activity showed significant associations with baseline ADMA concentration but not with the hypoxia-induced increase in ADMA in the present study. 

We previously noted a gradual decrease in plasma SDMA concentration in individuals exposed to chronic intermittent hypoxia [8]. The major enzyme metabolizing SDMA is AGXT2 [10]. We therefore speculate that AGXT2 expression and/or activity may be up-regulated in chronic hypoxia, to compensate deficient DDAH activity. This mechanism would limit the elevation of ADMA in hypoxia, and it could explain the observed decrement in SDMA concentration during hypoxia. The present data further support this hypothesis by showing that the reduction of SDMA was limited to carriers of the major allele of AGXT2 rs37369, while heterozygous individuals or those homozygous for the minor allele experienced no decrease in SDMA. The minor allele of this genetic polymorphisms has previously been associated with a reduced AGXT2 activity [25,26]. We therefore propose that AGXT2 is up-regulated in hypoxia, which, however, does not lead to enhanced enzymatic activity in carriers of the minor allele. 

The role of DDAH2 in ADMA metabolism has remained more controversial than that of DDAH1. DDAH2 is mainly expressed in cells co-expressing endothelial NO synthase, i.e., heart, placenta, kidneys, and lungs [27]. Knockdown of DDAH2 in rats impaired endothelium-dependent vasodilation but did not change circulating ADMA concentration [28]. Likewise, genetic deletion of DDAH2 in mice causes elevated ADMA concentration in myocardium and kidneys but no change in circulating ADMA, impaired endothelium-dependent relaxation to acetylcholine and elevated systolic blood pressure [29]. The DDAH2 SNP that we analysed is located 1151 base pairs upstream of the translational start site of the DDAH2 gene, raising the possibility that it may affect promoter binding. Maas and co-workers [14] genotyped 783 individuals from a population-based cohort for SNPs in DDAH1 and DDAH2 and reported that DDAH2 rs805304 was not associated with baseline circulating ADMA, but that homozygous carriers of the major allele had an odds ratio of 1.70 (1.22–2.36) for the presence of hypertension. 

Taken together, the present data in combination with previous studies suggest that presence of the major or minor allele of this genetic polymorphism determines regulation of DDAH2 gene expression in hypoxia and other pathophysiological conditions, while not affecting baseline ADMA plasma concentration. In line with this, we recently observed compensatory up-regulation of DDAH2 mRNA and protein expression in DDAH1 knockout mice exposed to chronic hypoxia [6].

The present study provides additional evidence to support a functional role of the L-arginine—dimethylarginine—NO pathway in the pathophysiology of health consequences arising from exposure to chronic intermittent hypoxia. A variety of single nucleotide polymorphisms in genes relating to nitric oxide bioavailability were significantly associated with high-altitude pulmonary hypertension. These data support a role of the L-arginine—dimethylarginine—NO pathway in the long-term adaptation to repetitive exposure towards high altitude, in line with previous reports comparing yaks with cattle [30] and lama with sheep [31]. Persistent high-altitude pulmonary hypertension during chronic hypoxia and, importantly, also during chronic intermittent hypoxia, may lead to serious health consequences by causing right ventricular hypertrophy and, finally, right heart failure [1].

The present study was performed in young Chilean males undergoing military service after ethical approval and individual informed consent was obtained. None of the study participants was exposed to chronic intermittent hypoxia solely for purposes of this study; rather, the study participants were observed at regular intervals during their daily and weekly routines with no interventions except for venous blood sampling, health questionnaires, and echocardiography after six months in a subgroup. This cohort offered the advantage that additional health hazards impacting on mining workers at high altitude (e.g., dust, heavy physical work) did not affect participants of this study. Furthermore, the investigated cohort was naïve to high altitude. 

Our study has several limitations. It is limited to young Chilean men with a high prevalence of obesity and smoking; therefore, we cannot extrapolate our data to elderly individuals with pulmonary diseases or to the general population. The relatively small sample size must be considered in cautiously interpreting associations of the minor alleles with clinical status. Additional molecular analyses will be required to further elucidate the exact mechanisms underlying this regulation, the specific transcription factors involved, and the molecular signalling pathways linking hypoxia to the observed changes in the L-arginine—dimethylarginine—NO pathway. Furthermore, in this study mean pulmonary arterial pressure was estimated using echocardiography with its inherent, well-recognized limitations. Although high-altitude experts generally agree with the definition of high-altitude pulmonary hypertension used in this study [18], it deviates from the general cut-off value for pulmonary arterial pressure defining pulmonary arterial hypertension (≥20 mm Hg) [32]; an evidence-based, revised definition of pulmonary hypertension at high altitude is clearly needed to better define this condition and the pathophysiology related to it [33]. Finally, we suppose that single nucleotide polymorphisms analysed in this study directly or indirectly interfere with nitric oxide metabolism; however, we have not measured nitric oxide metabolites in plasma and therefore cannot prove this.

## 5. Conclusions

In conclusion, this study provides genetic evidence for differential roles of the three enzymes involved in dimethylarginine metabolism during exposure to chronic hypoxia. Taken together with previous observations, the present data support the notion that DDAH1 is the major enzyme metabolizing ADMA in physiological conditions, while both DDAH2 and AGXT2 are regulated in conditions in which DDAH1 expression and/or activity is impaired, therefore playing important roles in back-up enzymatic pathways for ADMA degradation.

## Figures and Tables

**Table 1:** Baseline characteristics of the study participants and effect of chronic intermittent hypoxia on selected variables.

Variable	Units	Baseline	6 Months
Age	years	18.0 (18.0–19.0)	n.a.
Male sex	*n* (%)	69 (100)	n.a.
Smoker	*n* (%)	35 (50.7)	n.a.
Height	m	1.73 (1.68–1.76)	n.a.
Weight	kg	69.0 (63.0–78.0)	n.a.
BMI	kg/m^2^	24.2 (21.6–26.1)	n.a.
Arterial oxygen saturation	%	98.0 (98.0–98.5)	91.0 (87.5–93.0) **
Systolic blood pressure	mm Hg	110 (105–120)	110 (100–112.5)
Diastolic blood pressure	mm Hg	70.0 (70.0–75.0)	70.0 (65.0–80.0)
Heart rate	L/min	71.5 (65.5–78.3)	74.5 (68.5–82.0)
Haematocrit	%	45.0 (44.0–46.0)	48.5 (46.6.–49.9) **
Haemoglobin	mg/dL	14.9 (14.5–15.4)	16.0 (15.7–16.4) **
ADMA	µmol/L	0.68 (0.60–0.75)	0.72 (0.62–0.78) *
SDMA	µmol/L	0.67 (0.59–0.75)	0.58 (0.53–0.66) *
L-Arginine	µmol/L	16.6 (11.9–23.4)	29.6 (21.1–34.6) **

Data are median [25th percentile–75th percentile] unless indicated otherwise. Physiological and biochemical parameters and biomarker levels presented here were measured at baseline under sea level conditions. Abbreviations: BMI, body mass index; ADMA, asymmetric dimethylarginine; SDMA, symmetric dimethylarginine. * *p* < 0.05, ** *p* < 0.001 versus baseline.

**Table 2:** Single nucleotide polymorphisms studied.

Gene/SNP	Major/Minor Allele	Expected Allele Frequency ^#^	Measured Allele Frequency	*p*
NOS3				
rs1799983	G/T	0.785/0.215	0.790/0.210	ns
rs2070744	T/C	0.742/0.258	0.717/0.283	ns
rs891512	G/A	0.860/0.140	0.862/0.138	ns
DDAH1				
rs1241321	A/G	0.671/0.239	0.674/0.326	ns
rs480414	G/A	0.735/0.265	0.812/0.188	ns
rs233112	T/C	0.666/0.334	0.652/0.348	ns
DDAH2				
rs805304	T/G	0.549/0.451	0.601/0.399	ns
rs2272592	C/T	0.925/0.075	0.913/0.087	ns
ARG1				
rs2246012	T/C	0.751/0.249	0.775/0.225	ns
rs2781667	C/T	0.524/0.475	0.580/0.420	ns
ARG2				
rs3742879	A/G	0.767/0.233	0.775/0.225	ns
rs3759757	G/C	0.635/0.365	0.659/0.341	ns
AGXT2				
rs37369	C/T	0.676/0.324	0.703/0.297	ns
rs16899974	C/A	0.739/0.261	0.710/0.290	ns
PRMT1				
rs10415880	G/A	0.801/0.199	0.833/0.167	ns
rs975484	C/G	0.682/0.318	0.768/0.232	0.0434

^#^ Expected allele frequencies were obtained from the LDLink database [19,21] of the National Cancer Institute (https://www.cancer.gov (accessed on 20 November 2021)) and are based on the 1000 Genomes Project [22,23]. Data are given as relative frequencies, representing allelic distribution of every polymorphism as fractions of 1. *p* values were calculated using the actual haplotype counts within the Mixed American population according to LDLink and within our cohort of Chilean individuals, respectively. Abbreviations: SNP, single nucleotide polymorphism; NOS3, endothelial nitric oxide synthase; DDAH, dimethylarginine dimethylaminohydrolase; ARG, arginase; AGXT, alanine glyoxylate aminotransferase; PRMT, protein arginine methyltransferase.

## Data Availability

The data presented in this study are available on request from the corresponding author.
